# Congenital diaphragmatic hernias: from genes to mechanisms to therapies

**DOI:** 10.1242/dmm.028365

**Published:** 2017-08-01

**Authors:** Gabrielle Kardon, Kate G. Ackerman, David J. McCulley, Yufeng Shen, Julia Wynn, Linshan Shang, Eric Bogenschutz, Xin Sun, Wendy K. Chung

**Affiliations:** 1 Department of Human Genetics, University of Utah, Salt Lake City, UT 84112, USA; 2 Departments of Pediatrics (Critical Care) and Biomedical Genetics, University of Rochester Medical Center, Rochester, NY 14642, USA; 3 Department of Pediatrics, University of Wisconsin, Madison, WI 53792, USA; 4 Department of Systems Biology, Columbia University Medical Center, New York, NY 10032, USA; 5 Departments of Pediatrics, Columbia University Medical Center, New York, NY 10032, USA; 6 Department of Pediatrics, University of California, San Diego, La Jolla, CA 92093, USA; 7 Department of Medicine, Columbia University Medical Center, New York, NY 10032, USA

**Keywords:** Structural birth defects, Congenital diaphragmatic hernia (CDH), Diaphragm, Pulmonary hypoplasia, Pulmonary hypertension, Congenital heart disease (CHD), Genetics

## Abstract

Congenital diaphragmatic hernias (CDHs) and structural anomalies of the diaphragm are a common class of congenital birth defects that are associated with significant morbidity and mortality due to associated pulmonary hypoplasia, pulmonary hypertension and heart failure. In ∼30% of CDH patients, genomic analyses have identified a range of genetic defects, including chromosomal anomalies, copy number variants and sequence variants. The affected genes identified in CDH patients include transcription factors, such as *GATA4*, *ZFPM2*, *NR2F2* and *WT1*, and signaling pathway components, including members of the retinoic acid pathway. Mutations in these genes affect diaphragm development and can have pleiotropic effects on pulmonary and cardiac development. New therapies, including fetal endoscopic tracheal occlusion and prenatal transplacental fetal treatments, aim to normalize lung development and pulmonary vascular tone to prevent and treat lung hypoplasia and pulmonary hypertension, respectively. Studies of the association between particular genetic mutations and clinical outcomes should allow us to better understand the origin of this birth defect and to improve our ability to predict and identify patients most likely to benefit from specialized treatment strategies.

## Abstract (precis)

**Summary:** In this Review, Kardon et al. discuss the complex etiology of congenital diaphragmatic hernia, as revealed by comprehensive genomic analyses and modeling in mice, and highlight the need for new therapies to treat this developmental disorder.

## Introduction

Birth defects represent some of the most complex challenges in medicine. A structural problem in one organ can have associated comorbidities in many other systems because defects in one organ, such as the heart or diaphragm, can affect the development or function of another, such as the lungs or because the structural defect is part of a multisystem genetic syndrome (Abman et al., 2015). The heterogeneous etiology and range of co-morbid conditions of many common structural defects can hamper progress in their treatment. Clinical care for such complex congenital conditions often involves multi-disciplinary teams, drawn from a variety of specialties, to tackle the clinical challenges these conditions pose (Khokha et al., 2015).

The diaphragm is a skeletal muscle that normally separates the thoracic and abdominal cavities and is essential for respiration. Defects in diaphragm development are common, occurring in about 1 in 3000 births worldwide (Stege et al., 2003), and are associated with complicated and often devastating clinical outcomes. Individuals with congenital diaphragmatic hernias (CDHs, see Box 1, Glossary) have weakened or incompletely developed diaphragms that allow the contents of the abdomen to herniate into the thoracic cavity, thereby mechanically impeding lung and heart development. CDH patients often have pulmonary hypoplasia and pulmonary hypertension (see Box 1, Glossary) and present with cardiopulmonary failure at birth. CDH is diagnosed prenatally in ∼50% of cases (Stege et al., 2003). Prenatally, clinicians quantify lung volume to predict prognosis. Unfortunately, our ability to predict outcomes and to treat CDH is hindered by technical challenges and by differences in the degree of diaphragm, heart, and lung dysfunction among CDH patients. Recently, advances have been made in our understanding of the genetic pathways that regulate normal diaphragm development and the genetic mutations that lead to CDH. These insights have revealed the complexity of this disorder, as mutations in multiple genes and defects in different cellular mechanisms can result in CDH. Furthermore, some CDH-associated genetic mutations affect not only development of the diaphragm but also directly affect the development of other organs, such as the heart and lungs. Thus multi-factorial genetic, cellular, and mechanical mechanisms underlie the complex pathogenesis of CDH and ultimately lead to highly variable disease outcomes.

**Box 1. Glossary**

**Bochdalek hernia:** A developmental diaphragmatic defect that involves the lumbocostal triangle at the posterior-lateral chest wall; clinicians often use this term to imprecisely describe many types of CDH.

**Congenital diaphragmatic hernia (CDH):** Diaphragmatic hernias that involve the posterior-lateral diaphragm and present in newborns with respiratory distress.

**Ductus arteriosus:** Fetal anatomical shunt between the pulmonary and systemic circulation. In CDH patients with severe pulmonary hypertension, the ductus arteriosus is often kept open by treatment with a prostaglandin E1 (PGE1) infusion to decrease the workload on the right ventricle.

**Eventration:** Abnormally thin diaphragm usually associated with muscularization defects.

**Fetal endoscopic tracheal occlusion (FETO):** Fetal surgical procedure in which the trachea is occluded (usually with a balloon) to cause fetal lung fluid accumulation, which induces the stretching and possibly accelerated development of the lungs.

**Morgagni hernia:** A hernia that occurs in the anterior diaphragm just posterior (usually just posterior-lateral) to the sternum. These hernias are not usually discovered in newborns, and are not usually associated with pulmonary hypoplasia.

**Pleuroperitoneal folds (PPFs):** Transient pyramidal structures at the base of the embryonic thoracic region that give rise to the muscle connective tissue and central tendon of the diaphragm.

**Pulmonary hypertension:** High blood pressure in the vascular system of the lungs. CDH patients often have cardiopulmonary failure that is marked by severe pulmonary hypertension.

**Pulmonary hypoplasia:** Lungs that are small due to abnormal development, as seen in patients with CDH.

Recent advances, including decreased sequencing costs and the ability to make mouse models more easily, have allowed researchers to study CDH more effectively, leading to new insights into the genetic and developmental contributors to CDH, which we review here. We also discuss the cardiopulmonary consequences of CDH, and the available and emerging treatment options.

## Overview of CDH

**Fig. 1.**
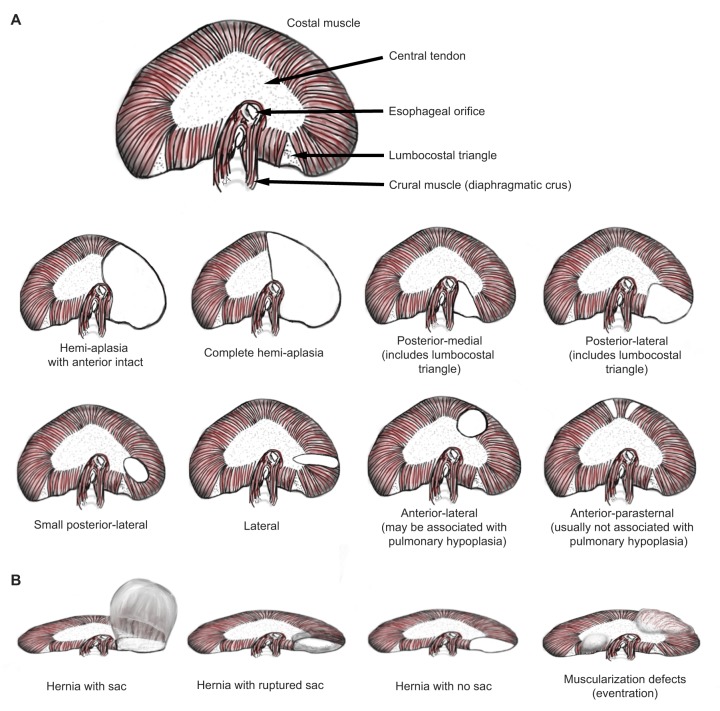
**Anatomy of the human diaphragm at birth and types of diaphragmatic defects.** Anatomy and localization of diaphragm defects depicted from a cranial view, with anterior (this is called the ventral region in the embryo) at top and posterior (dorsal region in the embryo) at bottom. (A) A normal diaphragm (top). Different types of diaphragm defects (below). The first row of defects shows different types of Bochdalek hernias. The second row shows other types of hernias, including anterior lateral and anterior parasternal defects that are considered to be Morgagni hernias. (B) Different diaphragm defects from a posterior view. Drawings by K. Ackerman.

The diaphragm consists of two muscle domains. The costal diaphragm is a domed sheet of muscle composed of a radial array of muscle fibers, which extends from the ribs to a central tendon (Fig. 1). The crural diaphragm, which is located in the posterior region (developmental biologists call this the dorsal region), attaches to the vertebrae and surrounds the esophagus and aorta. CDH mainly results from defects in development of the costal diaphragm. Classically, diaphragm defects are classified as being either Bochdalek or Morgagni types (Irish et al., 1996) (Fig. 1). Bochdalek hernias (see Box 1, Glossary) occur in the lumbocostal triangle at the posterolateral (dorsolateral, in the parlance of developmental biologists) wall of the thorax and are associated with pulmonary hypoplasia and respiratory failure at birth. Morgagni hernias (see Box 1, Glossary) are caused by defects in the anterior (ventral, in the parlance of developmental biologists) diaphragm and are usually not associated with pulmonary hypoplasia (Al-Salem, 2007). They can be discovered incidentally or when associated with respiratory infection. A systematic analysis of patients with lethal diaphragm defects shows that there are many other phenotypic variants (Ackerman et al., 2012). Defects in posterior (including posteromedial and posterolateral), lateral, or sometimes anterolateral regions are all associated with respiratory disease. Defects can also involve the entire hemi-diaphragm, although the most anterior portion usually remains intact. The central tendon of the diaphragm is rarely involved (Pober, 2007).

Severe CDH that presents at birth is usually associated with co-morbidities in other organs (Slavotinek, 2014). Approximately 20% of CDH patients are estimated to have a structural cardiac anomaly (Menon et al., 2013). Lung defects occur in most CDH cases diagnosed at birth; these defects include small lungs and abnormal tracheal branching and lobation. The lung defects arise from the herniated tissue physically hindering the developing lungs (Adzick et al., 1985) and from abnormalities in lung development itself (Ackerman et al., 2005; Keijzer et al., 2000). The relative contribution of either mechanism is unknown. Since the most difficult medical management challenge is posed by pulmonary hypertension, it is important to understand how the pulmonary vascular system and cardiac function are developmentally altered in CDH. CDH patients can also have problems in other organ systems, making this a systemic disease. These include gastroesophageal reflux, feeding problems, and failure to thrive, as well as skeletal deformities, such as scoliosis. There is also an elevated prevalence of neurodevelopmental and behavioral issues (Wynn et al., 2013).

Currently, the clinical management of CHD largely consists of supportive care with surgical repair of the diaphragm postnatally. This care includes respiratory support with positive ‘gentle’ ventilator pressures, modulation of the pulmonary vascular system, measures to prevent right-heart failure by allowing for patent ductus arteriosus shunting (Box 1, glossary) when pulmonary vascular pressures are high, and other routine intensive care support. Some patients do not tolerate the switch from fetal circulation and require extra corporeal membrane oxygenation (ECMO). Despite such intensive support, the mortality rate for CDH remains high, and there is a desperate need for new, specific therapies to be developed that target CDH pathophysiology (Reiss et al., 2010). An important avenue for the discovery of new therapies is the identification of genes and molecular pathways that are disrupted in CDH.

## Genetic basis of CDH

Both environmental and genetic factors are thought to contribute to the etiology of CDH. To date, genetic causes have been identified in ∼30% of cases (Russell et al., 2012; Yu et al., 2015). There are few studies of the heritability of CDH because its high mortality has generally prohibited the analysis of familial cases of vertical transmission. In one analysis of 203 cases, 100% (5/5) of monozygotic twins were discordant for CDH, and the recurrence rate among siblings was 0.7% (1/149; Pober et al., 2005), supporting the hypothesis that *de novo* mutations constitute a significant fraction of the genetic alterations predisposing babies to developing CDH. Mutations in particular CDH-associated genes or chromosomal regions are incompletely penetrant for CDH (Longoni et al., 2012) and cause a range of diaphragm defects (Yu et al., 2013). In addition, some CDH-associated genes, especially those that encode transcription factors, such as GATA binding protein 6 (*GATA6*) and nuclear receptor subfamily 2 group F (*NR2F2*, also known as *COUPTFII*), have also been associated with other congenital anomalies, including congenital heart disease and pancreatic agenesis (see Table 1). CDH mutations can have pleiotropic effects and expressivity that varies between affected individuals (Chao et al., 2015; High et al., 2016; Wang et al., 2012; Yu et al., 2014).

**Table 1.**
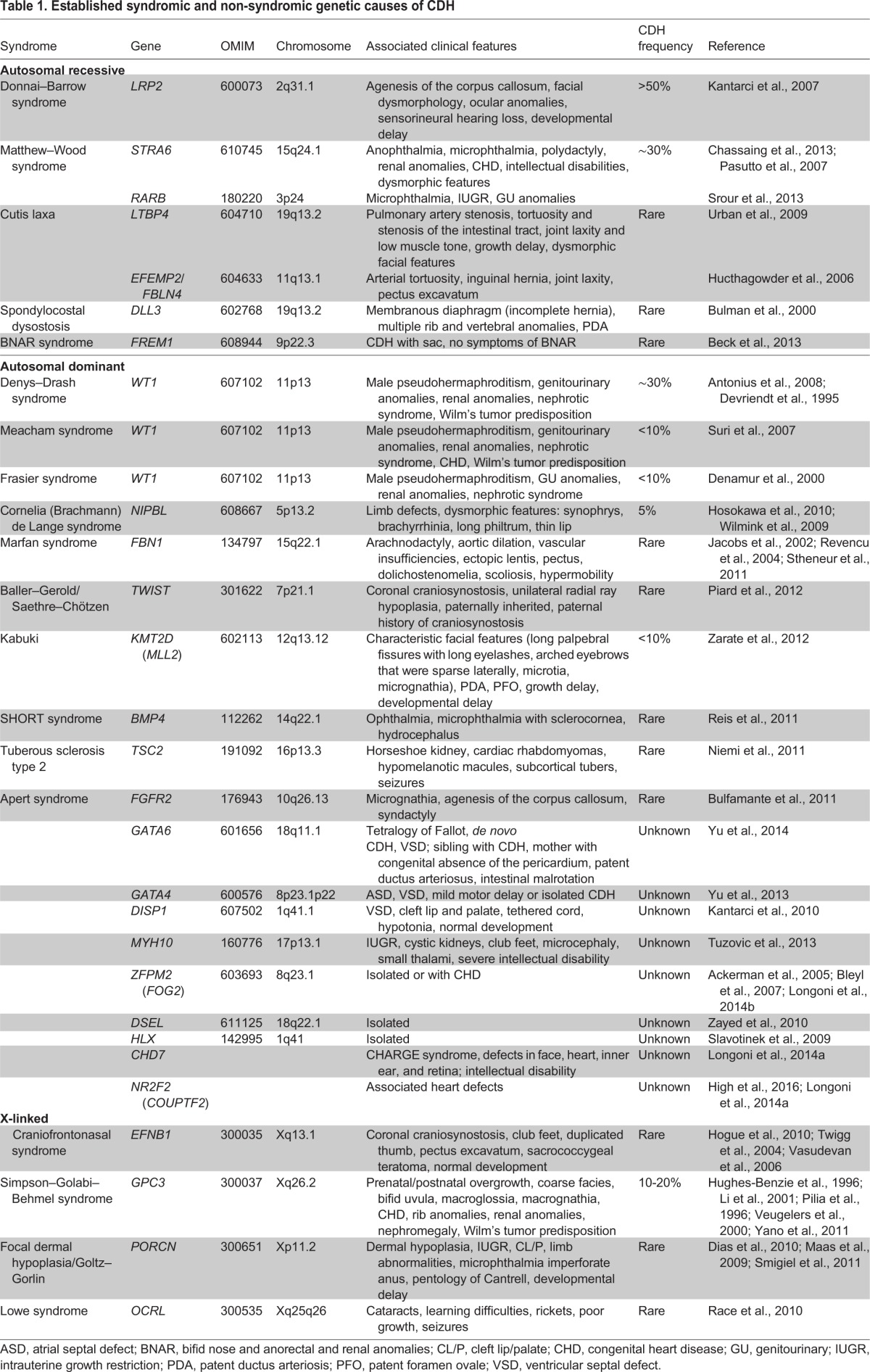
**Established syndromic and non-syndromic genetic causes of CDH**

The genetic etiology of CDH is highly heterogeneous and includes aneuploidies, cytogenetic rearrangements, copy number variants (CNVs) and single-gene mutations. CDH can be caused by chromosomal anomalies (Brady et al., 2011; Ding et al., 2005; Holder et al., 2007). After ultrasound identification of CDH, it is now routine to perform prenatal karyotype and/or chromosome microarray on fetal cells sampled during amniocentesis to identify genetic anomalies (Scott et al., 2007; Slavotinek et al., 2006). Chromosome aneuploidies, large chromosome CNVs, and complex chromosome rearrangements are observed in 10-35% of CDH cases (Beck et al., 2008; Howe et al., 1996; Pober et al., 2005; Samangaya et al., 2012; Tonks et al., 2004; Wynn et al., 2014). Anomalies of multiple chromosomes are associated with CDH (Table 1). The most frequent CDH-associated aneuploidies include trisomy 18, trisomy 13, trisomy 21, and less frequently trisomy 9, trisomy 16, trisomy 22, mosaic trisomy 2, Turner syndrome (45, X) and trisomy X (46, XXX) (Beck et al., 2008; Wynn et al., 2014). Pathogenic CNVs are observed in 3.5-13% of CDH cases (Srisupundit et al., 2010; Wat et al., 2011; Wynn et al., 2014; Yu et al., 2012), and most frequently include tetrasomy 12p, 15q26.1-26.2 deletion, 8p23.1 deletion, 1q41-42 deletion and 4p16 deletion (Biggio et al., 2004; Shimokawa et al., 2005; Youssoufian et al., 1988). Chromosomal anomalies are most frequently associated with CDH cases that occur with other comorbid conditions, and there are over 70 syndromes in which CDH is described as a clinical feature. Mendelian syndromes associated with CDH that have an identified genetic basis are highlighted in Table 1.

Until recently, most of the individual genes implicated in CDH were identified through the characterization of mutant mouse models (Table 2) and through the analysis of recurrent chromosomal anomalies in CDH patients (Table 1). Genes implicated in CDH include *GATA4* (Jay et al., 2007; Merrell et al., 2015; Yu et al., 2013), *GATA6* (Yu et al., 2014), zinc finger protein multitype 2 (*ZFPM2*; also known as *FOG2*; Ackerman et al., 2005), *NR2F2* (High et al., 2016), Wilms tumor 1 (*WT1*) (Paris et al., 2015; Scott et al., 2005; Suri et al., 2007), FRAS1-related extracellular matrix protein (*FREM1*; Beck et al., 2013), fibrillin 1 (*FBN1*; Beck et al., 2015), myosin heavy chain 10 (*MYH10*; Tuzovic et al., 2013), dispatched 1 (*DISP1*; Kantarci et al., 2010), Delta like 3 (*DLL3*; Bulman et al., 2000), low density lipoprotein-related protein 2 (*LRP2*; Kantarci et al., 2007), *STRA6* (Pasutto et al., 2007) and *FBLN4* (Hucthagowder et al., 2006). Vitamin A and the retinoid signaling pathway are also reported to play key roles in CDH (Greer et al., 2003; Kling and Schnitzer, 2007). Notably, many of the genes associated with CDH (see Table 1) have also been associated with other congenital anomalies or neurodevelopmental disorders (Deciphering Developmental Disorders Study, 2015; Homsy et al., 2015; Zaidi et al., 2013).

**Table 2.**
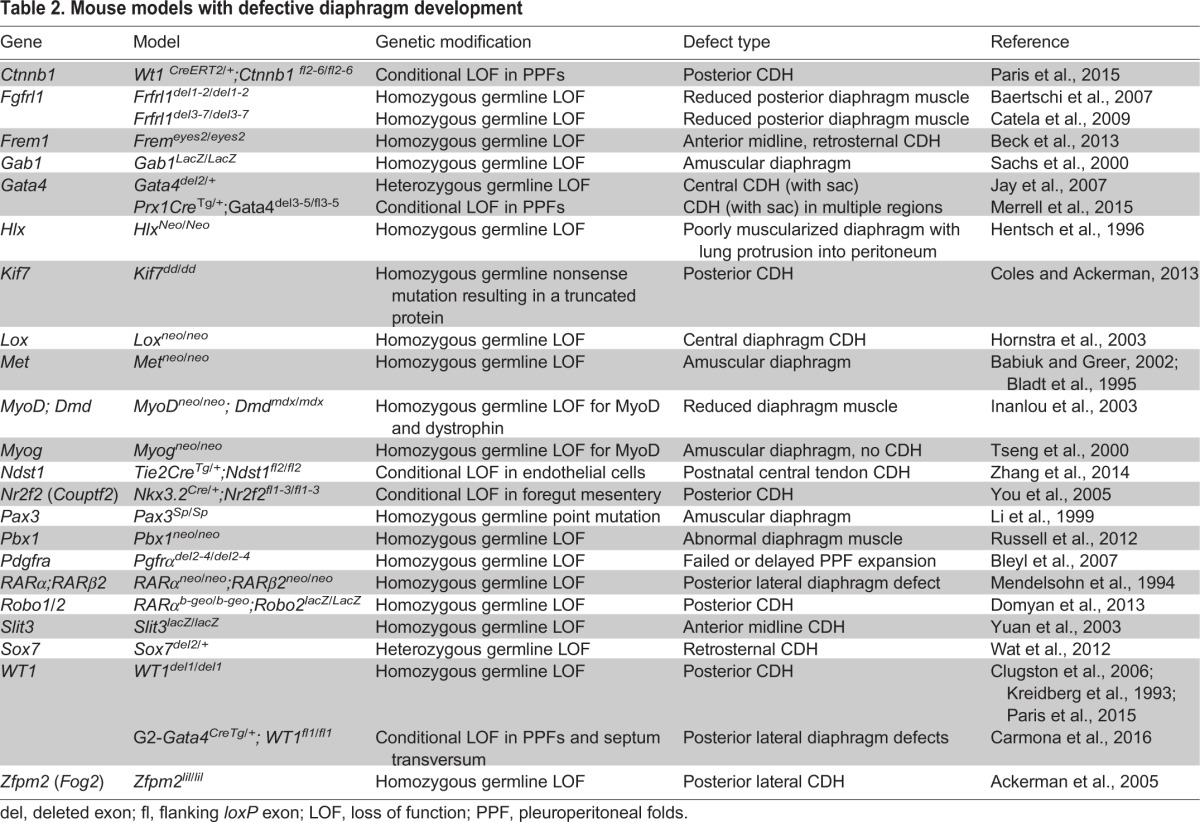
**Mouse models with defective diaphragm development**

Two genes encoding transcription factors, *GATA4* and *NR2F2*, have been implicated by multiple studies to cause CDH. *GATA4* is located in the chromosomal region 8p23.1, and microdeletions of 8p23.1 and single-nucleotide mutations in *GATA4* are associated with CDH (Arrington et al., 2012; Longoni et al., 2012; Wat et al., 2009). In addition, coding mutations in *GATA4* have been implicated in isolated, familial CDH (Yu et al., 2013). Furthermore, genetic studies of *Gata4* in mice have definitively demonstrated that *Gata4* is required for normal lung and diaphragm development, and that *Gata4* loss-of-function mutations cause CDH (Ackerman et al., 2007; Jay et al., 2007; Merrell et al., 2015). *NR2F2* encodes a retinoic acid-activated nuclear receptor (Kruse et al., 2008), and it maps to 15q26.1 – a chromosomal region that is strongly associated with CDH (Klaassens et al., 2005). A recent study identified a *de novo* frame-shift mutation in *NR2F2* in a CDH patient with an atrial septal defect (High et al., 2016). In addition, conditional deletion of *Nr2f2* in mice demonstrates that loss of *Nr2f2* gives rise to CDH (You et al., 2005).

CDH is usually a sporadic condition but familial cases have been described (Slavotinek, 2014), and genetic causes underlying both familial and sporadic CDH have identified (Table 1). Mutations in *LRP2*, *ZFPM2* and *GATA4* have been found in familial cases of CDH (Kantarci et al., 2007; Longoni et al., 2014b; Yu et al., 2013). In sporadic cases, *de novo* single nucleotide variants (SNVs), insertions and deletions (indels), or large CNVs are expected to contribute significantly to the etiology of these cases. Studies of trios of unaffected parents with CDH-affected children identified, via cytogenetic and microarray analyses, *de novo* pathogenic CNVs (affecting one or more genes) in 4% of CDH patients (Yu et al., 2012). This represents a CNV occurrence rate similar to that of other congenital anomalies (Greenway et al., 2009; Sanna-Cherchi et al., 2012). Recent advances in whole exome sequencing (WES) and in whole genome sequencing (WGS) have greatly facilitated the detection of *de novo* (as well as inherited) SNVs, indels and CNVs. Using WES on 39 trios of unaffected parents and CDH children, the DHREAMS study (http://www.cdhgenetics.com/dhreams-study.cfm) identified an excess burden of *de novo* variants that are likely to disrupt or be deleterious to genes highly expressed during diaphragm development (Yu et al., 2015). Based on these data, 15% of sporadic non-isolated CDH cases were estimated to be attributable to gene-disrupting or deleterious missense mutations (Yu et al., 2015). WES data from another large CDH cohort identified 11% of cases with ultra-rare, likely disruptive, gene variants (Longoni et al., 2014a). Interestingly, in these two large WES studies, no single gene was found that had two or more damaging *de novo* or rare variants, indicating the potentially large number of genes that might contribute to CDH. This is also the case for congenital heart disease, for which an estimated ∼400 risk genes are believed to contribute to the disease through *de novo* coding mutations (Homsy et al., 2015; Zaidi et al., 2013). Since we hypothesize that a similarly large number of genes are likely to contribute to CDH, a thousand trios would need to be analyzed to identify 20 genes with recurrent mutations in two or more cases. Such recurrent mutations are necessary to confidently impute the role of these genes in CDH.

The genetic etiology of CDH has been identified for less than half of CDH cases worldwide (Brady et al., 2011; Holder et al., 2007; Longoni et al., 2014a; Pober et al., 1993; Samangaya et al., 2012; Wynn et al., 2014; Yu et al., 2015, 2012), and in our experience (L.S., Y.S., J.W., W.K.C.) is known for less than 30% of cases. WGS analysis is an important tool for discovering the genetic basis of CDH; in contrast to WES, it can detect noncoding variants, as well as complex indels and structural variants. Discordant identical twin data raise the possibility that somatic mutations or epigenetic abnormalities might contribute to CDH development (Pober et al., 2005), and these areas have been under-explored. Somatic mutations can be identified using high-depth WGS, and epigenetic changes are detectable by methods such as bisulfite sequencing. The future use of WGS should allow both sequence and structural variations, and inherited and somatic mutations, to be detected in a single test. Future comprehensive analyses of genomic DNA and RNA sequences, epigenetic marks and chromatin modifications of diaphragm tissue should enable the evaluation of the potential role of somatic mutations, regulatory variants and epigenetic changes in CDH.

Our increased ability to identify the genetic etiology of CDH should also provide new clinical opportunities. Advances in the quality and accessibility of prenatal ultrasound screening enable early CDH diagnosis, and genetic data might provide better prognostic information to guide clinical management. As genetic diagnoses are made, we can begin to address the question of whether any particular CDH case is expected to be isolated or whether the implicated genetic mutation can be expected to have pleiotropic effects on other organs, such as the heart, lungs and brain. A key to understanding the effects of these genetic mutations is to determine the function of these genes in development of the normal diaphragm and CDH, as well as possible roles in development of the heart and lungs.

## Diaphragm and CDH development

**Fig. 2.**
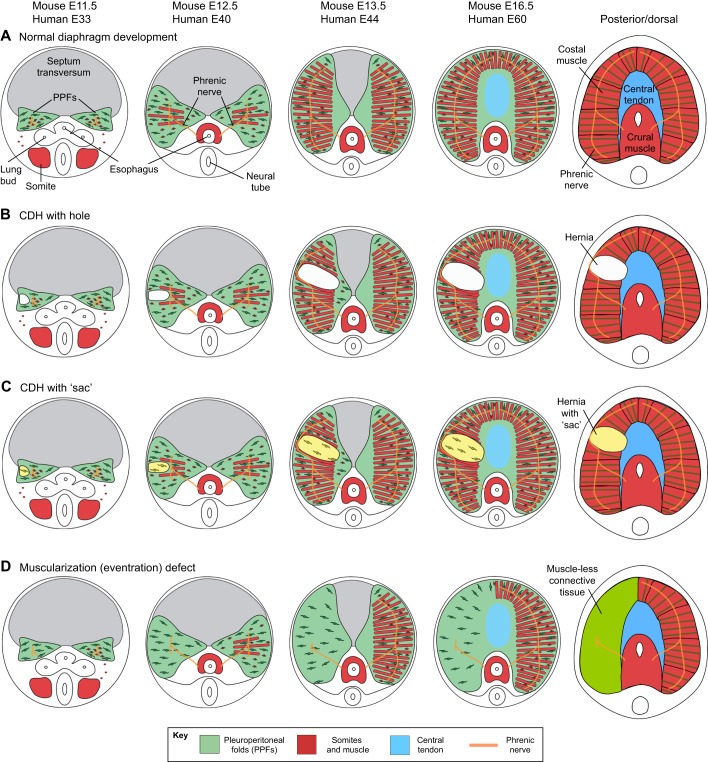
**Development of the diaphragm and diaphragm defects.** (A) Normal development of the mouse diaphragm. Pleuroperitoneal folds (PPFs; green) give rise to muscle connective tissue and to the central tendon. Somites (red) give rise to muscle. Septum transversum (gray) is proposed to give rise to cells of the central tendon, but this has not been formally tested. The stage of embryonic development is indicated above each representative image, for mouse and humans. (B) Development of CDH with a hole (featuring loss of muscle and connective tissue), which allows abdominal contents to herniate into the thoracic cavity. This is generally thought to result from defects in the PPF cells. (C) Development of CDH with a muscle-less connective tissue ‘sac’ covering herniated tissue. In one case, this has been demonstrated to result from genetic defects in the PPFs, which in turn lead to the development of muscle-less patches that allow herniation (Merrell et al., 2015). Note that the size and location of defects can vary. (D) Development of diaphragm that lacks muscle on the left side. Muscle-less hemi-diaphragm can also develop on the right side. Note that for all diaphragm defects, the size and location of the defect can vary. Drawings by G. Kardon.

The diaphragm's muscle, connective tissue, central tendon and phrenic nerve become integrated into a functional structure during development (Fig. 2). The diaphragm develops from several embryonic tissues, beginning with the development [at embryonic day (E)8.5 in mouse, E18 in human] of the septum transversum, a thin mesodermal sheet positioned above the liver (Dunwoodie et al., 1998). The septum serves as the initial barrier between the thoracic and abdominal cavities (Iritani, 1984). Next to develop are the pleuroperitoneal folds (PPFs, see Box 1, Glossary), two transient pyramidal structures that lie on either side of the esophagus and are adjacent to the underlying septum (Allan and Greer, 1997; Iritani, 1984; Merrell et al., 2015). The PPF fibroblasts proliferate and spread posteriorly (dorsally) and anteriorly (ventrally) across the septum and ultimately give rise to the muscle connective tissue and central tendon (Merrell et al., 2015). The diaphragm's costal muscle develops from muscle progenitors that delaminate from the somites and migrate into the PPFs (Dietrich et al., 1999; Babiuk et al., 2003). As the PPFs spread, the muscle cells are carried throughout the diaphragm and differentiate into the radial array of costal myofibers (Merrell et al., 2015). Finally, the diaphragm is innervated by the phrenic nerve, which originates from the cervical region of the neural tube (Allan and Greer, 1997). Genetic manipulation of mice has revealed that the PPFs control the development of the costal muscle and the overall morphogenesis of the costal diaphragm (Merrell et al., 2015). Thus, the PPFs ensure that muscle, connective tissue, and tendon develop in tandem and are functionally integrated.

CDH encompasses multiple phenotypes that result from different defects in diaphragm development. Most commonly, CDH presents as a herniation of the abdominal contents into the thoracic cavity through a hole in the diaphragm (Fig. 1B and Fig. 2B) (Ackerman et al., 2012). Abdominal tissue can also herniate through localized diaphragm regions that contain connective tissue but no muscle, resulting in hernias surrounded by connective tissue ‘sacs’ (Fig. 1B and Fig. 2C). Hernias, with or without sacs, can occur either through the muscled regions of the diaphragm or the central tendon (Ackerman et al., 2012). Another type of defect (often called an eventration by clinicians, see Box 1, Glossary) is a muscularization defect, whereby a large region is composed of muscle-less connective tissue that ascends abnormally high into the thoracic cavity (Fig. 1B and Fig. 2D).

The mechanisms that regulate how these different defects develop are just beginning to be elucidated, largely by *in vivo* studies of mice that have been pharmacologically or genetically manipulated (Table 2). The formation of a hole where diaphragm muscle normally develops (Fig. 2B) is generally attributed to defects in the development of the PPFs, which give rise to the muscle connective tissue and the central tendon; decreased proliferation, increased apoptosis, migration failure, and alteration in differentiation of PPF fibroblasts have all been implicated as playing a role (Clugston et al., 2010b; Coles and Ackerman, 2013; Paris et al., 2015). Genetic defects in the septum transversum are also a potential source of CDH (Carmona et al., 2016). A developmental mechanism that causes hernias with sacs (Fig. 2C) has been recently elucidated through the genetic inactivation of *Gata4* in mice (Merrell et al., 2015). This study showed that hernias with sacs form via the development of localized regions that lack muscle progenitors (via their decreased proliferation and increased apoptosis), and this leads to amuscular patches that are mechanically weaker than the surrounding muscularized diaphragm, resulting in herniation (Merrell et al., 2015). The formation of entirely muscle-less diaphragms or hemi-diaphragms (Fig. 2D) can result from the failed migration of muscle progenitors from the somites into the PPFs (Babiuk and Greer, 2002). Interactions with tissues neighboring the diaphragm might also contribute to the development of CDH. For instance, central tendon hernias have been proposed to arise when the diaphragm fails to separate from the underlying liver during development (Yuan et al., 2003). More recently, aberrant foregut morphogenesis in the mouse has been shown to cause diaphragm defects that allow herniation (Domyan et al., 2013). Thus, there appear to be multiple developmental scenarios that can lead to diaphragm defects.

An important question for CDH research to address is whether CDH cases share a common cell of origin and whether defects arise in common molecular pathways or processes. Genetic and gene expression studies suggest that PPF cells are most often the cell of origin. Several studies have shown that CDH-associated genes are specifically expressed in PPF fibroblasts (Clugston et al., 2008; Merrell et al., 2015; Paris et al., 2015). For instance, *GATA4* and its co-factor, *ZFPM2*, are both expressed in the PPFs, and mutations in both genes have been implicated by multiple human and mouse studies to cause CDH (Ackerman et al., 2005; Arrington et al., 2012; Brady et al., 2014; Jay et al., 2007; Longoni et al., 2012, 2014b; Merrell et al., 2015; Yu et al., 2013). Conditional mutagenesis studies in mice have provided strong evidence that *Gata4* functions in PPF fibroblasts and that *Gata4-*deficient fibroblasts cause non-cell-autonomous effects on neighboring myogenic progenitors (Merrell et al., 2015). Conditional mutagenesis of β-catenin (*Ctnnb1*) in mice has also implicated mesothelial cells associated with PPFs as a cellular source of CDH (Paris et al., 2015). Genetic defects that affect the diaphragm muscle or central tendon might also give rise to CDH, although this remains to be tested. It also remains unclear as to whether the many genetic mutations and chromosomal abnormalities associated with CDH affect only a small number of common downstream pathways or processes or whether multiple mechanisms are involved. For example, the retinoic acid pathway has been implicated by many studies to be defective in CDH (Clugston et al., 2010a; Merrell and Kardon, 2013; Noble et al., 2007). Defects in the maintenance of the extracellular matrix of the connective tissue of the diaphragm's muscle and central tendon can also contribute to CDH (Hornstra et al., 2003; Lin et al., 2006). The discovery of common downstream pathways or processes that lead to CDH is likely to be vital for developing general therapeutic targets to promote diaphragm muscle growth.

The complexities of early diaphragm development pose a major barrier to discovering whether common pathways or processes underlie the development of CDH. While three embryonic structures (somites, PPFs and septum transversum) play vital roles in diaphragm development, other structures, such as the posthepatic mesenchymal plate (PHMP), could be important (Carmona et al., 2016; Iritani, 1984; Mayer et al., 2011). There is confusion in the literature as to the identity of many of these structures, particularly the PPFs and the PHMP. This confusion arises because of the three-dimensional complexity of the region and the lack of unique molecular markers and infrequent use of genetic reagents (i.e. *Cre* alleles) to mark and follow the fate of these structures during development. Most studies have relied on analyses of histologically stained sections whose orientations are not uniform. Future progress will be enhanced by the whole-mount analysis of embryos in which structures that give rise to the diaphragm are labeled with specific antibodies or *Cre* alleles.

## Cardiopulmonary consequences of CDH

Although abnormal diaphragm development is the defining hallmark of CDH, the high rate of mortality and the long-term disability of CDH patients are largely due to abnormal lung and pulmonary vasculature development and function, which cause lung hypoplasia and pulmonary hypertension (Box 2) (Dillon et al., 2004; Lusk et al., 2015). In CDH patients who survive the neonatal period, lung volume measurements normalize within the first few weeks of life; however, airway and perfusion defects persist, indicating that lung and pulmonary vascular structures remain abnormal (Ijsselstijn et al., 1997; Spoel et al., 2016; Stefanutti et al., 2004; Trachsel et al., 2005; Wohl et al., 1977). Traditionally, it was assumed that the lung and pulmonary vascular defects of CDH patients are caused by mechanical compression brought about by the abnormally positioned abdominal organs. More recently, a dual-hit hypothesis has been proposed in which lung defects are due to a combination of mechanical compression and primary lung defects that occur independently of the diaphragm defect (Keijzer et al., 2000).

**Box 2. Cardiopulmonary consequences of CDH**

**Lung defects**

Premature arrest of airway branching

Decreased number and size of alveoli

Long-term obstructive and restrictive lung disease

Airway hyper-reactivity

Abnormal diffusion capacity

**Pulmonary vascular defects**

Pulmonary hypertension

Premature arrest of arterial branching

Decreased proximal vessel size

Decreased alveolar capillary density

Increased vascular smooth muscle thickness

Ectopic positioning of vascular smooth muscle

Decreased response to vasodilating cues

Increased circulating vasoconstrictors

**Cardiac defects**

Increased incidence of congenital heart disease

Decreased size of left-sided structures

Ductal and intra-cardiac shunt

Right heart dysfunction/failure

Normal lung development depends on the regulation of mechanical forces within the chest and lungs (Harding, 1997; Joe et al., 1997; Kitterman, 1996). However, the mechanisms by which decreased intrathoracic volume causes the premature arrest of lung branching and the overall reduction in alveoli seen in CDH patients are not well understood. Within the developing lung mesenchyme, alveolar myofibroblasts play a central role in alveologenesis and detect changes in extracellular matrix stiffness via activation of the mechanosensory transient receptor vanilloid 4 (TRPV4) channels (Boström et al., 1996; Lindahl et al., 1997; McGowan et al., 2008; Rahaman et al., 2014). The activation or inhibition of TRPV4 channels alters the differentiation of lung myofibroblasts by affecting TGFβ-dependent actomyosin remodeling and the nuclear localization of the transcriptional regulator, myocardin-related transcription factor (MRTF-A; Rahaman et al., 2014). Within the developing lung epithelium, changes in mechanical strain are detected by lung epithelial integrins α6 and α1, which activate the tumor necrosis factor-α enzyme (TACE)-mediated release of the EGFR (epidermal growth factor receptor) ligands heparin binding (HB)-EGF and TGFα (Zaidi et al., 2013). This, in turn, regulates the differentiation of alveolar type II epithelial cells, which are required for surfactant production (Zaidi et al., 2013). Improving our understanding of how these and other mechanosensory mechanisms are involved in lung development could guide strategies to promote compensatory lung growth in CDH patients. By fine-tuning the timing and mechanical properties of interventions such as fetal tracheal occlusion, clinicians may be able to improve the pulmonary outcomes.

In support of the dual-hit hypothesis, there is increasing evidence that some CDH patients have primary defects of the lung and pulmonary vasculature. A recent study of CDH patients with lesions that cause decreased thoracic space in the developing fetus demonstrated that, in comparison to patients with congenital pulmonary airway malformation, whose lungs are distorted by cysts that develop within the tissue, CDH patients have more severe lung and pulmonary vascular defects (Derderian et al., 2016). This finding suggests that mechanical compression alone is not solely responsible for the lung and pulmonary vascular defects seen in this disorder. Recent genetic analyses of CDH patients, and complementary experiments performed in animal models, suggest that genetic defects that impair diaphragm development might also directly impair lung and pulmonary vascular development and function. As mentioned above, mutations in *GATA4* and *ZFPM2* in CDH patients and mice lead to defects in diaphragm development and CDH (Ackerman et al., 2005; Arrington et al., 2012; Brady et al., 2014; Jay et al., 2007; Longoni et al., 2012, 2014b; Merrell et al., 2015; Yu et al., 2013). Loss of function of either *Gata4* or *Zfpm2* in mice also directly results in lung abnormalities, including decreased lung growth, abnormal branching, and changes in lung mesenchyme and in epithelial cell differentiation (Ackerman et al., 2005, 2007; Jay et al., 2007). *ZFPM2* and *NR2F2* are both implicated in human CDH and are regulated by retinoic acid signaling, which in turn plays an important role in lung development (Malpel et al., 2000; Marquez and Cardoso, 2016).

The pulmonary neuroendocrine cell plays a unique role in the pulmonary pathophysiology of CDH. This is a rare epithelial cell type in the lung that preferentially resides at airway branching points (Kuo and Krasnow, 2015; Noguchi et al., 2015). These cells are believed to function as sensors of mechanical forces, of oxygen concentration, and of other airway signals (Cutz et al., 2013). They also produce proteins, including secreted neuropeptides and amines, that can stimulate lung growth (Sakai et al., 2014). Two such proteins, bombesin and ghrelin, have been reported to regulate the level of retinoic acid receptors, which in turn increased lung growth in explant culture (Pereira-Terra et al., 2015b). A more direct role for pulmonary neuroendocrine cells in CDH has been recently demonstrated in a study using Roundabout (*Robo*) and *Slit* mouse mutants. ROBO and SLIT proteins are best known for their function in axon guidance and angiogenesis (Blockus and Chédotal, 2016). Both ROBO and SLIT have been implicated in human CDH (Branchfield et al., 2016; Kantarci and Donahoe, 2007; Longoni et al., 2014a). In mouse *Robo* and *Slit* mutants, pulmonary neuroendocrine cells are abnormally unclustered, and this resulted in up-regulation of neuropeptide secretion, increased immune cell infiltration, destruction of normal ECM, and in the subsequent simplification of the alveolar septae of the lung (Branchfield et al., 2016). The regulation of pulmonary neuroendocrine cells and their secreted products in lung morphogenesis by CDH-implicated genes shows how such genes can directly affect lung development and pathophysiology in CDH. Further investigation of how lung development is altered by the genetic mutations found in CDH patients could in the future guide clinical interventions that promote lung growth and function. For example, blocking neuropeptides or immune cell infiltration might prevent the adverse action of immune cells on the ECM and alveolar septae. Such interventions would need to be carried out immediately after birth to prevent permanent structural damage to the developing lungs.

Multiple mechanisms underlie the development of pulmonary hypertension in CDH patients. The cumulative effect of the observed lung abnormalities results in an overall reduction in the number of lung vessels. Similar to the lung abnormalities, pulmonary vascular defects tend to be more severe on the same side as the hernia, suggesting that mechanical compression plays a role (Geggel et al., 1985; Kitagawa et al., 1971). In addition, several factors that affect either lung vessel formation (vascular endothelial growth factor, VEGF) or pulmonary vascular smooth muscle relaxation after birth (endothelial adhesion molecules ICAM-1, ELAM-1, VCAM-1, thromboxane B2, endothelin-1, monocyte chemotactic factor 1) are abnormally regulated in CDH patients (Bos et al., 1990; Fleck et al., 2013; Keller et al., 2010; Kobayashi et al., 2004; Nakayama et al., 1992; Okawada et al., 2007; Patel et al., 2015; Shehata et al., 1999). The mechanisms responsible for the abnormal expression of these factors are not clear, but the genetic mutations that impair diaphragm development might also lead to the aberrant transcriptional regulation of these factors. A better understanding of the genetic defects that contribute to pulmonary hypertension in CDH patients is vitally important for our ability to improve patient survival. These insights might also help to explain why some patients fail to respond to pulmonary hypertension medication, and could highlight new approaches to therapy.

In addition to lung and pulmonary vascular defects, CDH patients have a higher incidence of abnormal cardiac development (Box 2). Altered blood flow through the embryonic heart can lead to the abnormal development of the pulmonary arteries and the left-sided structures of the heart (Allan et al., 1996; Byrne et al., 2015; Schwartz et al., 1994; Siebert et al., 1984; Thebaud et al., 1997; VanderWall et al., 1997; Vogel et al., 2010). There is also an increased frequency of major structural malformations in the hearts of CDH patients (Graziano and Congenital Diaphragmatic Hernia Study Group, 2005; Lin et al., 2005). In addition, many of the same genes mutated in CDH also play a critical role in cardiac development or function. Thus patients with mutations in genes such as *EPHA3*, *EZH2*, *GATA4*, *GATA6*, *PBX1*, *ROBO1*, *SEMA3A*, *TWIST1* and *ZFPM2* often have diaphragm and heart defects (Chang et al., 2008; Delgado-Olguin et al., 2012; Garg et al., 2003; Ieda et al., 2007; Kodo et al., 2009; Kuo et al., 1997; Lepore et al., 2006; Molkentin et al., 1997; Mommersteeg et al., 2015; Shelton and Yutzey, 2008; Stankunas et al., 2008; Stephen et al., 2007; Svensson et al., 2000; Tevosian et al., 2000; Vincentz et al., 2008; Zhou et al., 2009). The combination of congenital heart disease and CDH significantly increases mortality in these patients and complicates their long-term outcome (Cohen et al., 2002; Gray et al., 2013; Graziano and Congenital Diaphragmatic Hernia Study Group, 2005).

Thus, although CDH arises from defects in diaphragm development, the cardiopulmonary consequences are the principal cause of the morbidity and mortality associated with CDH. Treatments for CDH patients have largely focused on these cardiopulmonary issues, as outlined below.

## Available and emerging treatment strategies

The survival of CDH patients has significantly improved in the past 20 years, largely due to changes in respiratory management. Respiratory care now focuses on gentle ventilation and applies guidelines to reduce variation in care (Badillo and Gingalewski, 2014; Puligandla et al., 2015; Reiss et al., 2010). The goal is to protect the lungs by decreasing injury caused by mechanical ventilation or by high oxygen concentrations. More recent investigations have focused on approaches that actively enhance lung growth during fetal development, while minimizing pulmonary hypertension. Many fetal and early postnatal interventions that focus on lung growth and development have been studied in humans (Deprest et al., 2014; Grivell et al., 2015) and in animal models (Eastwood et al., 2015).

Fetal endoscopic tracheal occlusion (FETO, see Box 1, Glossary) is one of the most thoroughly studied interventions that aim to promote lung growth in CDH patients. FETO takes advantage of fluid production within the fetal lung to induce stretch and growth by preventing lung fluid from exiting the airway. Although FETO has evolved significantly, it is reserved for patients with low estimated fetal lung volumes who have the greatest risk of mortality. The current approach includes insertion of a balloon to occlude the trachea at 26-28 weeks of gestation, followed by removal of the balloon at 34 weeks. Despite the advance made in FETO, several questions remain about its use, including the optimal timing of the occlusion, how to allow for the cycling of pressure within the lung, and how to identify patients most likely to benefit despite the associated risks, which include fetal demise, preterm delivery, fetal or maternal infection, fetal or maternal blood loss, and damage to the fetal airway (Al-Maary et al., 2016). Improved knowledge of the CDH-associated genetic defects that impact the mechanosensory mechanisms of lung development will help determine which patients are more likely to respond to FETO.

How to best identify the patient population most likely to benefit from a procedure is a key issue when considering a new approach to treatment. Currently, plans for postnatal care and decisions about fetal therapy for CDH patients are based on the evaluation of lung size as assessed by fetal ultrasound or MRI. Although these measurements provide some information about risk, fetal lung size does not always correlate with postnatal cardiopulmonary function. Because no single measurement has emerged as being the best at predicting postnatal outcome, a combination of approaches might offer better predictive power (Le et al., 2012). Using a combined approach that incorporates morphological, physiological and genetic information from fetuses with CDH might help to identify the population most likely to benefit from currently available fetal treatments.

Genomic analysis could play a role in determining those patients most likely to respond to specific fetal or postnatal therapies. Fetal chromosome microarray analysis is frequently offered to expecting parents once a CDH diagnosis is made (Scott et al., 2007; Slavotinek et al., 2006), and fetal exome sequencing is now available clinically (Carss et al., 2014; Drury et al., 2015). A CDH-specific array was developed to identify CNVs previously implicated in CDH or genes associated with diaphragm or lung development (Srisupundit et al., 2010). From a screen of 79 patients with isolated CDH, the investigators described three patients with CNVs who underwent fetal tracheal occlusion, all of whom died postnatally due to lung hypoplasia and pulmonary hypertension (Srisupundit et al., 2010). It is possible that the CNVs (8p deletion resulting in a heterozygous loss of *GATA4*, an *EFNB1* gene duplication, and a mosaic trisomy 2) limited the response of these patients to tracheal occlusion. However, reaching such a conclusion is difficult without knowing the baseline mortality rate in patients undergoing this procedure in the absence of pathogenic CNVs (Srisupundit et al., 2010). In the future, fetal exome or genome sequence analysis, correlated with clinical outcomes, might help to guide decisions about which patients will benefit from FETO.

Given that the currently available tools for assessing fetal anatomy and for fetal genetic testing lack sensitivity and specificity, the identification of additional biomarkers that correlate with disease severity is an important CDH research goal. Recently, it was demonstrated that response to FETO was significantly improved in CDH patients who have higher expression levels of the microRNA, miR-200b, present in the tracheal fluid collected at the time of balloon removal (Pereira-Terra et al., 2015a). This microRNA is expressed in both lung epithelial and endothelial cells, and it functions to decrease TGFβ-mediated activation of SMAD signaling proteins in the lung epithelium, a recently described negative consequence of FETO (Pereira-Terra et al., 2015a; Vuckovic et al., 2016). It is unclear if the observed variation in miR-200b expression was due to differences in the regulation of miR-200b inherent to the patient or to small variations in the FETO procedure itself. Perhaps more significantly, the expression of the microRNA miR-10a was found to be lower at the time of balloon insertion in patients who had a more vigorous response to FETO (Pereira-Terra et al., 2015a). This difference in miR-10a expression prior to the intervention might serve as a marker to identify those patients who are most likely to respond to FETO.

Because of the risks associated with FETO, identifying transplacental fetal treatment methods that encourage lung growth and development while reducing pulmonary hypertension is important. Studies in humans and in animal models suggest that vitamin A and retinoic acid play an important role in the pathogenesis of CDH and impact both diaphragm and lung development (Gallot et al., 2005; Greer et al., 2003; Major et al., 1998; Marquez and Cardoso, 2016). In the nitrofen-induced rat model of CDH (in which retinal dehydrogenase-2 is inhibited), fetal supplementation with vitamin A or retinoic acid reduces the incidence of CDH and improves lung hypoplasia, lung development and vascular abnormalities (Babiuk et al., 2004; Baptista et al., 2005; Montedonico et al., 2008; Schmidt et al., 2012; Thebaud et al., 2001, 1999). In surgical models of CDH, retinoic acid supplementation normalized alveolar epithelial differentiation in rabbits (Gallot et al., 2008), while vitamin A treatment reduced ventilator-induced lung injury and improved lung morphology and function in lambs (Lewis et al., 2011, 2006). A recent review (Eastwood et al., 2015) highlights many of the difficulties associated with translating these findings to CDH patients, including the concern about the teratogenic effects of retinoic acid treatment during fetal development. Further analysis of the downstream targets of retinoic acid signaling during lung and pulmonary vascular development might help to identify therapeutic targets to enhance lung growth, while reducing the risks associated with the global activation of this pathway.

Pulmonary hypertension in CDH patients is difficult to control, and advances that promote pulmonary vasodilation and decrease right ventricular afterload (pressure to eject blood out of the heart) can be life-saving. For example, it is now common practice to maintain an open ductus arteriosus (see see Box 1, Glossary) in postnatal CDH patients affected by severe pulmonary hypertension (see see Box 1, Glossary; Keller, 2012). In addition to postnatal treatment advances, many investigators have been working in animal models of CDH to identify fetal pharmacological strategies that reduce pulmonary hypertension. Phosphodeisterase-5 (PDE5) inhibitors, such as sildenafil or tadalafil are often used to treat persistent pulmonary hypertension in CDH patients after birth. Recently, these medications have also been demonstrated to reduce postnatal pulmonary hypertension when treatment is initiated during fetal development (Kattan et al., 2014; Lemus-Varela et al., 2014; Luong et al., 2011; Russo et al., 2016; Shue et al., 2014; Yamamoto et al., 2014). In rats with nitrofen-induced CDH, prenatal treatment with sildenafil, given to the pregnant mother, improved lung and pulmonary vascular development, increased the expression of VEGF and endothelial nitric oxide synthase (eNOS), and increased the postnatal response to the nitric oxide donor, DEANO (Luong et al., 2011). Importantly, this prenatal inhibition of PDE5 did not alter vascular or morphological development of the retina or brain (Luong et al., 2011). Subsequent studies in the nitrofen-induced rat model of CDH, and in rabbit and sheep surgical models, have demonstrated that prenatal treatment with sildenafil or with tadalafil effectively inhibits fetal phosphodiesterase-5, improves pulmonary vascular growth and development, reduces vascular smooth muscle remodeling and improves vascular smooth muscle reactivity (Kattan et al., 2014; Lemus-Varela et al., 2014; Russo et al., 2016; Shue et al., 2014; Yamamoto et al., 2014). These investigations demonstrate that prenatal treatment with PDE5 inhibitors can ameliorate many of the key morphological and physiological mechanisms of pulmonary hypertension that are associated with CDH. Several other prenatal pharmacological approaches have been used to decrease pulmonary hypertension in animal models of CDH, including treatment with prostacyclin analogs, angiotensin antagonists, vitamin A and steroids (Gonçalves et al., 2014; Nogueira-Silva et al., 2012; Okoye et al., 1998; Schmidt et al., 2013; Taira et al., 1998; Umeda et al., 2016). Although each of these medications has been demonstrated to reduce pulmonary hypertension when administered after birth, the goal is to minimize the risk of life-threatening pulmonary hypertension during the newborn period by starting the treatment prenatally. Further studies will be required to demonstrate the safety and efficacy of these treatments in human fetuses with CDH.

## Conclusion

Although we have made significant advances in improving the outcomes of CDH patients – through improved prenatal diagnosis and the use of interventions such as FETO and ECMO – the morbidity and mortality of CDH remains high, especially in patients who have other associated anomalies. Advances in genomics, coupled with functional studies in animal models, are increasingly identifying the causes of CDH in both familial and sporadic cases. Through these approaches, we are beginning to elucidate the mechanisms and molecular pathways that are responsible for diaphragm and lung development abnormalities in CDH patients. A key challenge will be to understand which molecular pathways are most commonly disrupted and contribute to diaphragm and lung defects in CDH. An additional challenge will be to understand what causes the phenotypic variability and different clinical outcomes of CDH patients (or their mouse model counterparts) who share the same genetic mutation. We believe that the combined efforts of clinical investigators and developmental biologists will lead to new insights into the etiology of CDH that will improve patient prognosis and care as well as identify future targets for therapy.
